# Protective Association of Tumor Necrosis Factor Superfamily 15 (TNFSF15) Polymorphic Haplotype with Ulcerative Colitis and Crohn's Disease in an Indian Population

**DOI:** 10.1371/journal.pone.0114665

**Published:** 2014-12-12

**Authors:** Kirankumar Baskaran, Srinivasan Pugazhendhi, Balakrishnan S. Ramakrishna

**Affiliations:** 1 Wellcome Trust Research Laboratory, Christian Medical College, Vellore 632 004, India; 2 SRM Institutes for Medical Science, 1 Jawaharlal Nehru Road, Vadapalani, Chennai 600 026, India; INSERM, France

## Abstract

**Background:**

Tumor necrosis factor superfamily (TNFSF) proteins are involved in the genesis of inflammatory bowel disease (IBD). We examined the association of seven single nucleotide polymorphisms (SNP) in the *TNFSF15* gene with Crohn's disease (CD) and ulcerative colitis (UC) in the Indian population.

**Methods:**

Seven SNPs in the *TNFSF15* gene (rs10114470, rs3810936, rs6478108, rs4263839, rs6478109, rs7848647 and rs7869487) were genotyped in 309 CD patients, 330 UC patients and 437 healthy controls using the Sequenom iPLEX MassArray platform. Disease associations were evaluated for allelotypes and for genotypes.

**Results:**

The minor T alleles and the TT genotypes of rs10114470 and rs3810936 were significantly protectively associated with both CD and UC. The CC genotype of rs6478108, AA genotype of rs4263839, the AA genotype of rs6478109, the TT genotype of rs7848647 and the CC genotype of rs7869487 were all protectively associated with CD but not with UC. Two haplotype blocks could be discerned, one where SNPs rs10114470 and rs3810936 were in tight LD (D′ = 0.8) and the other where rs6478108, rs4263839, rs6478109, rs7848647 and rs7869487 were in tight LD (D′ 0.92–1.00). The second block of haplotypes were not associated with CD or with UC. The first block of haplotypes was very significantly associated with both CD and UC.

**Conclusions:**

Strong associations exist between *TNFSF15* gene polymorphisms and IBD (both CD and UC) in the Indian population.

## Introduction

The inflammatory bowel diseases (IBD), comprising ulcerative colitis (UC) and Crohn's disease (CD) are characterized by chronic inflammation of the intestine and/or colon resulting from complex gene-environment interactions [1]. Exaggerated and inappropriate mucosal immune responses to constituents of the intestinal microbiota contribute to the genesis of IBD, and these are determined by complex poorly understood genetic factors that confer vulnerability [1], [2]. There are geographical variations in the incidence and prevalence of IBD, with rates being highest in Europe and North America and low in much of Asia including South Asia [3], [4]. It is clear that Indians and other South Asians do not lack the genetic background necessary for development of IBD. Studies in South Asian migrants to the United Kingdom and North America indicate that incidence and prevalence of IBD in second generation migrants approaches or exceeds that in the local population [5]–[7].

The genetic background that permits development of IBD is complex, probably involves multiple genes that each confer a relatively small increase in risk of IBD, and characterized by population specific differences. The nucleotide-binding oligomerization domain 2 (*NOD2*) was the first gene to be identified as conferring susceptibility to CD. This gene, located at the IBD1 locus on chromosome 16, was identified using linkage analyses and fine mapping approaches [8], [9]. Studies identified 3 polymorphisms (R702W, G908R and 1007fs) as the major *NOD2* mutations present in Western patients with CD [10].

*NOD2* gene mutations are rare or absent in certain populations, specifically in Indian and Japanese patients with CD [11], [12], [13]. Among the Japanese, mutations in the tumor necrosis factor super family 15 (*TNFSF15*) gene are associated with CD [13], [14]. This gene, located on Chromosome 9q33, encodes the protein Tumor Necrosis Factor-like ligand 1a (TL1A) which is a member of the TNF superfamily. The interaction of TL1A with Death Receptor 3 (DR3) modulates the functions of T cells, NK, and NKT cells and drives the inflammatory processes in several T-cell-dependent autoimmune diseases [15]. Single nucleotide polymorphisms (SNPs) within the *TNFSF15* gene expressed highly significant associations in a GWAS study involving 484 Japanese CD patients and 1097 controls. An intronic T>C polymorphism (tnfsf15_28) was the most significant marker identified in this Japanese cohort but this was found to be monomorphic when replicated in two UK cohorts. Five SNPs (rs3810936, rs6478108, rs6478109, rs7848647, rs7869487) were found to be polymorphic in both Japanese and UK cohorts which were further explored in consecutive studies. Three haplotypes were defined by these 5 markers with haplotype A showing positive association and haplotype B expressing protective effect with CD in the Japanese and UK population [14]. A Japanese study also identified five SNPs at the *TNFSF15* locus that were associated with CD but not UC [16]. On the other hand, genotyping of these reported SNPs in US patients failed to confirm an association of risk haplotype A with either CD or UC though a protective effect of haplotype B was found in selected population groups [17]. Three of the five well-known *TNFSF15* SNPs were analyzed in a UK study, of which two (rs3810936, rs7848647) showed association with CD. Haplotype analysis based on these two SNPs confirmed the presence of a risk haplotype [18]. Association of these two SNPs (and rs6478108) with CD has also been replicated in a Korean population [19]. A familial association study showed over-transmission of risk alleles and haplotypes of rs6478108 and rs7869487 SNPs to affected family members in CD but not in UC [20]. A meta analysis of three genome wide association studies (GWAS) identified *TNFSF15* as a susceptibility locus for CD with rs4263839 SNP showing significant association [21]. This SNP was further examined in an Italian cohort where it showed unequivocal association with both adult and pediatric CD, as well as with early onset UC [22].

A synergistic effect of TL1A, interleukin-12 and interleukin-18 has been shown to augment interferon-γ release in mucosal CD4 +ve T-cells and natural killer cells [23]. Thus *TNFSF15* via TL1A augments Th1 responses in the intestine and TL1A overexpression in lymphoid and myeloid cells has been shown to lead to spontaneous colitis in experimental animals [24]. Occurrence of variants in the *TNFSF15* gene may influence the expression and function of TNFSF15 (TL1A) which may eventually result in disproportionate immune response in the bowel and altered cytokine production as seen in Crohn's disease. The present study was undertaken to determine whether specific SNPs in the *TNFSF15* gene associated with CD or UC in Indian patients with IBD.

## Methods

### Participants

Patients with a diagnosis of CD or UC were recruited from the IBD clinic of the Christian Medical College, Vellore between 2003 and 2010. The diagnosis of CD and UC was based on clinical, imaging, and histopathological findings, and exclusion of infective causes [25], [26]. The clinical work up of the patients followed the usual protocol of the hospital for IBD patients. Patients with proven intestinal or extra-intestinal tuberculosis, and those who refused consent to participate, were excluded. Controls were recruited from unrelated apparently healthy individuals visiting the outpatient clinics for health check-up. They were screened for history of any chronic illness including tuberculosis, leprosy and autoimmune disease prior to inclusion. As there may be regional differences in prevalence of genetic polymorphisms, the patients and controls were matched for region and compared accordingly. Each of the three groups comprised participants from southern India or eastern India in a ratio of approximately 2∶5. The history and clinical details were recorded and samples of venous blood were obtained in EDTA-anti-coagulated venous blood. Informed written consent for all parts of the study including clinical details, collecting blood and genetic testing was obtained from all study subjects, from the participants themselves in the case of adults and from the parents in the case of minors. The Institutional Review Board of the Christian Medical College approved the study protocol and consent forms.

### Genotyping of single nucleotide polymorphisms (SNP) in the *TNFSF15* gene

Genomic DNA was isolated from anti-coagulated venous blood by salting out method, checked for quality and concentration, and stored at −80°C until analysis. Seven SNPs - rs3810936, rs6478108, rs6478109, rs7848647, rs7869487, rs4263839 and rs10114470 – in the *TNFSF15* gene were selected for genotyping. The first five have been previously associated with CD in Japanese [14], UK [14], Korean [19], & US Jewish patients [17], while the last two were recognized as susceptibility loci in GWAS studies performed in 2008 and 2013 respectively [21], [27].

Genotyping of *TNFSF15* polymorphisms was performed on the Sequenom MassArray iPLEX platform at NxGenBio Life Sciences, Delhi, in 639 patients and 437 controls. Briefly, this involved design and validation of primers for each SNP leading to locus-specific PCR amplification followed by locus-specific primer extension in which oligonucleotide sequences annealed immediately upstream of the polymorphic site being genotyped. The primer extension detected SNPs in amplified DNA where the primer and the amplified target DNA were incubated with mass-modified dideoxynucleotide terminators. Primer extension occurred according to the sequence of the variant site, resulting in incorporation of a single complementary mass-modified base. Through the use of matrix-assisted laser desorption ionization–time-of-flight mass spectrometry, the mass of the extended primer was determined. The primer's mass indicated the sequence and, therefore, the alleles present at the polymorphic site of interest. The genotype was automatically allocated using software (SpectroTYPER, Sequenom) based on the mass of the observed primers.

### Statistical analysis

The comparison of allele and genotype distributions among cases and controls was done using PLINK v. 1.07 (website: http://pngu.mgh.harvard.edu/purcell/plink/) [28]. Statistical significance was evaluated, and odds ratios (OR), and 95% confidence intervals (CI) were calculated. Linkage disequilibrium (LD) pairwise values, haplotype structure, and haplotype frequencies were determined using the Haploview software v. 4.2 [29]. Significance of difference between groups was analysed using Chi square test.

## Results

A total of 309 (196 male) CD patients, 330 (200 male) UC patients and 437 (284 male) healthy controls were included in this study. Eighty three of the CD participants, 90 of the UC participants and 149 control participants were from southern India and the remainder from eastern India. The median (range) age was 35 (10–70) years for CD patients, 34 (15–76) years for UC patients, and 37 (18–73) years for controls. Disease extent for CD patients was distributed as follows: Ileal 111, colonic 67, ileocolonic 112, isolated upper gastrointestinal 7, and upper gastrointestinal with another level 12. Of the CD patients, 211 had inflammatory disease, 69 had stricturing disease and 29 had penetrating disease. Of the UC patients, 50 had proctitis, 87 had left sided colitis and 193 had pancolitis.

Genotyping of seven TNFSF15 variants was carried out in cases and controls. None of the SNPs investigated deviated significantly from Hardy-Weinberg equilibrium in any of the study groups.

### *TNFSF15* polymorphisms in IBD and control groups

Comparison of allelotype and genotype frequencies between cases and controls was carried out to ascertain association of SNPs with IBD. Table 1 shows minor allele frequency (MAF) of each of the seven SNPs in the three study groups (control. CD and UC), along with the calculated OR and 95% CI. Table 2 shows the frequency of each of the genotypes of the seven SNPs in the three study groups.

**Table 1:** Allele frequencies of TNFSF15 SNPs in cases and controls in Indian population.

.Marker	Chromosome	Position	Risk allele	Major/Minor Allele	Controls MAF	CD			UC		
						MAF	p-value	OR (95% CI)	MAF	p-value	OR (95% CI)
rs10114470	9	116587593	C	C/T	0.346	0.287	0.01*	0.76 (0.61–0.95)	0.290	0.02*	0.77 (0.62–0.96)
rs3810936	9	116592706	C	C/T	0.353	0.289	0.01*	0.74 (0.59–0.93)	0.295	0.01*	0.76 (0.61–0.95)
rs6478108	9	116598524	T	T/C	0.263	0.245	0.44	0.91 (0.71–1.15)	0.248	0.51	0.92 (0.73–1.16)
rs4263839	9	116606261	G	G/A	0.260	0.227	0.15	0.83 (0.65–1.07)	0.238	0.32	0.88 (0.69–1.12)
rs6478109	9	116608587	G	G/A	0.259	0.225	0.12	0.82 (0.64–1.05)	0.232	0.21	0.86 (0.67–1.09)
rs7848647	9	116608867	C	C/T	0.256	0.228	0.22	0.85 (0.67–1.09)	0.228	0.21	0.85 (0.67–1.09)
rs7869487	9	116620735	T	T/C	0.229	0.212	0.42	0.90 (0.70–1.16)	0.215	0.50	0.92 (0.72–1.17)

*Indicates significant P value.

**Table 2:** Genotype distributions of TNFSF15 SNPs in cases and controls.

SNP	Genotype	Controls	CD		UC	
		n (%)	n (%)	P - value	n (%)	P - value
rs10114470	CC	185 (42.5)	154 (50)	0.05*	157 (47.5)	0.01*
	CT	199 (45.7)	128 (42)		154 (46.7)	
	TT	51 (11.7)	24 (8)		19 (5.8)	
rs3810936	CC	182 (42)	152 (50.3)	0.03*	156 (48)	0.02*
	CT	192 (45)	125 (41.4)		146 (45)	
	TT	56 (13)	25 (8.3)		23 (7)	
rs6478108	TT	241 (56)	166 (55)	0.01*	186 (57)	0.68
	TC	150 (35)	125 (41)		115 (36)	
	CC	38 (9)	12 (4)		23 (7)	
rs4263839	GG	237 (56)	170 (58)	0.01*	185 (58)	0.31
	GA	147 (35)	114 (39)		116 (36)	
	AA	36 (9)	10 (3)		18 (6)	
rs6478109	GG	243 (56.3)	177 (58.2)	0.01*	192 (59)	0.31
	GA	152 (35.3)	117 (38.5)		115 (35)	
	AA	36 (8.4)	10 (3.3)		18 (6)	
rs7848647	CC	246 (57)	176 (58)	0.02*	194 (60)	0.32
	CT	149 (35)	117 (38)		112 (34)	
	TT	36 (8)	11 (4)		18 (6)	
rs7869487	TT	262 (61)	184 (61)	0.05*	203 (62)	0.77
	TC	140 (32)	111 (36)		104 (32)	
	CC	29 (7)	9 (3)		18 (6)	

Values shown are n (%). *Indicates significant P value.

#### rs10114470

The minor T allele of rs10114470 was associated with protection from both CD (OR 0.76, 95% CI 0.61–0.95) and UC (OR 0.77, 95% CI 0.62–0.96). The MAF was lower in CD (0.287) & UC (0.290) compared to controls having higher frequency of 0.346. This association was statistically significant yielding P- values of 0.01 for CD and 0.02 for UC. In keeping with this, the TT genotype of this SNP showed a higher frequency of 11.7 in control group compared to CD and UC patients who had lower frequencies of 8 and 5.8 respectively (P = 0.05 for CD and P = 0.01 for UC).

#### rs3810936

The T allele of rs3810936 showed a significant protective association with IBD. The MAF for CD and UC were 0.289 and 0.295 respectively, compared with controls who had higher frequency of 0.353 (P<0.01 for both CD and UC individually compared to control). The corresponding OR (95% CI) for this comparison was 0.74 (0.59–0.93) for CD and 0.76 (0.61–0.95) for UC. The protective association of rs3810936 with IBD was further confirmed on the basis of genotype frequency where the TT occurred with a frequency of 8.3% in CD and 7% in UC compared to controls who had a frequency of 13% (P = 0.03 for CD and P = 0.02 for UC).

#### rs6478108

There was no significant difference in MAF between cases and controls. However the CC genotype showed a significant protective association with CD but not UC.

#### rs7869487

Again there was no significant difference in MAF between cases and controls, but the CC genotype showed a significant protective association with CD.

#### rs4263839

There was no significant difference in MAF between cases and controls. The AA genotype showed a significant protective association with CD but not UC.

#### rs6478109

There was no significant difference in MAF between cases and controls. Again, the AA genotype showed a significant protective association with CD but not UC.

#### rs7848647

There was no significant difference in MAF between cases and controls. However, the TT genotype showed a significant protective association with CD but not UC (Table 2).

#### Haplotype analysis

**Figure 1:**
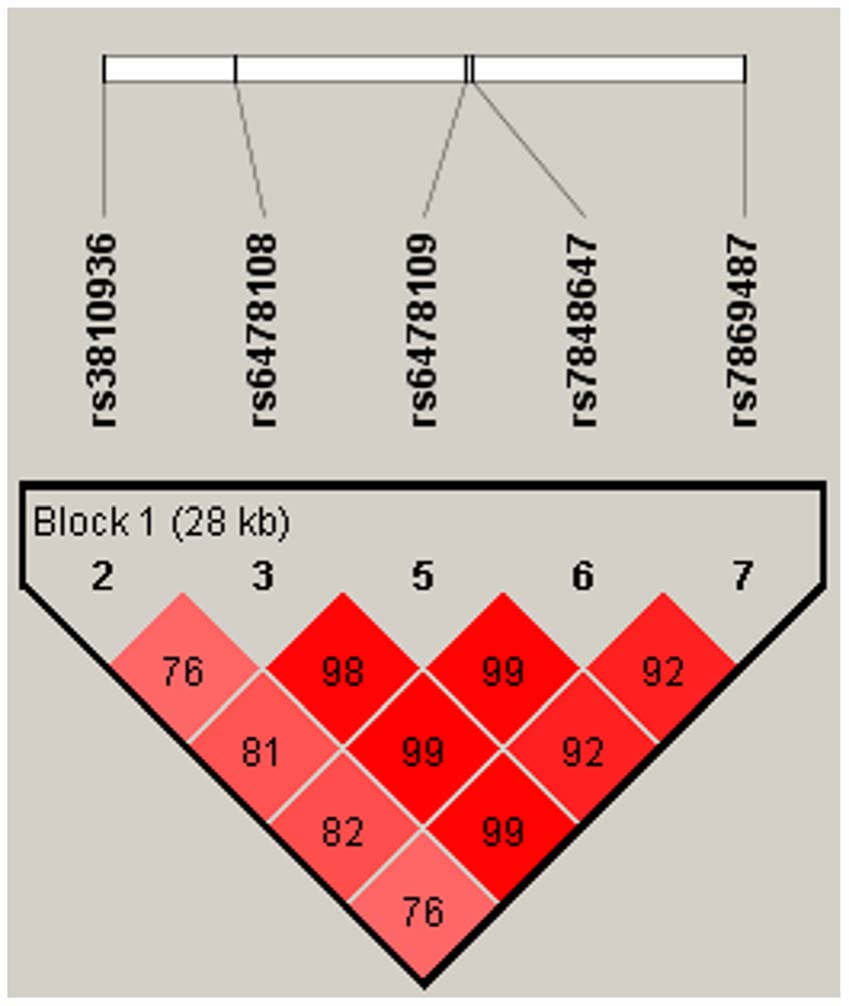
LD pattern between Five TNFSF15 SNPs. rs3810936, rs6478108, rs6478109, rs7848647, rs7869487 were in tight LD (D′ = 0.76–0.99) and were organized in single haplotype block.

Initially only five of the seven genotyped SNPs (rs38910936, rs6478108, rs6478109, rs7848647 & rs7869487) were used to construct six haplotypes (A–F) (Table 3, Fig. 1). All the 5 SNPs were organized in a single haplotype block. However LD among four SNPs (rs6478108, rs6478109, rs7848647 & rs7869487) was almost complete (D′ ranging from 0.92 to 0.99), while rs3810936 was in weak LD with rest of the four SNPs (D′ ranging from 0.76 to 0.82), The haplotype block structure of the five SNPs is shown in Table 3. Estimation of haplotype frequencies were done in case and controls separately in order to characterize the haplotypes of these five polymorphisms. Haplotype A, containing the risk alleles of all five SNPs, was significantly associated with IBD (Table 4). The carrier frequency for haplotype A was 0.66 in CD and 0.65 in UC, and was significantly less (0.60) in controls (P = 0.01 and 0.03 for CD and UC respectively). On the other hand, haplotype C had a protective association with IBD, with a frequency of 0.08 in CD and 0.09 in UC compared to 0.12 in controls (P = 0.02 for CD and 0.03 for UC). The remaining haplotypes were devoid of significant differences in their frequencies between cases and controls.

**Table 3:** Structure of haplotypes A-F constructed using only five TNFSF15 SNPs.

Haplotype	rs3810936	rs6478108	rs6478109	rs7848647	rs7869487
A	C	T	G	C	T
B	T	C	A	T	C
C	T	T	G	C	T
D	T	C	A	T	T
E	C	C	A	T	C
F	C	C	G	C	C

**Table 4:** Haplotype A-F frequency estimates in controls and IBD patients.

Haplotype	Control	CD	P -value	UC	P -value
A	0.60	0.66	0.01*	0.65	0.03*
B	0.19	0.17	0.21	0.17	0.22
C	0.12	0.08	0.02*	0.09	0.03*
D	0.02	0.02	0.97	0.02	0.85
E	0.02	0.02	0.87	0.02	0.97
F	0.006	0.01	0.08	0.01	0.10

*Indicates significant P value.

**Figure 2:**
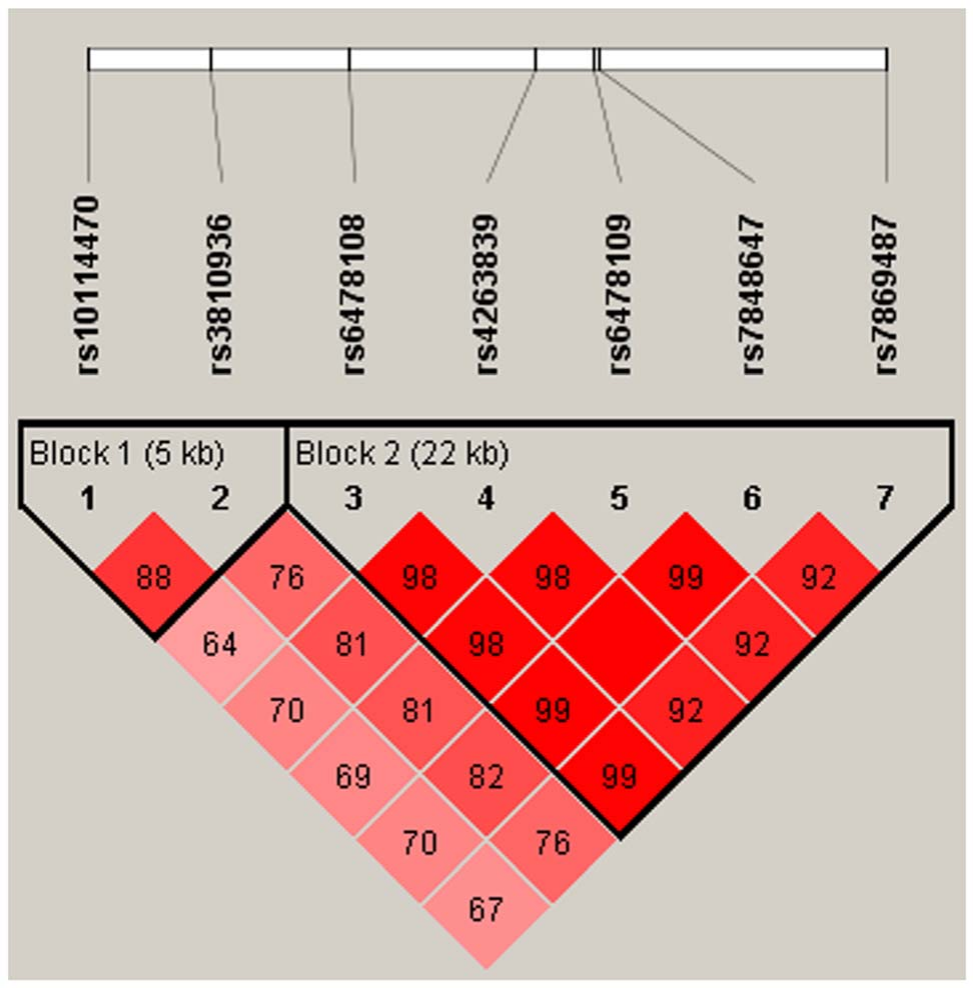
LD pattern between Seven TNFSF15 SNPs. rs10114470 & rs3810936 being in tight LD (D′ = 0.8) were organized in Block 1 and rs6478108, rs4263839, rs6478109, rs7848647, rs7869487 being in tight LD (D′ = 0.92–1.0) were organized in Block 2 respectively.

Inclusion of all seven SNPs in haplotype analysis generated a different block structure (Table 5, Fig. 2) where SNPs rs10114470 and rs3810936 were in tight LD (D′ = 0.8) and were organized in block 1. The remaining 5 SNPs were organized in block 2 whose LD ranged from 0.92–1.0. Seven TNFSF15 SNPs formed 8 haplotypes (G-N) with four in each block respectively. On evaluating the differences in haplotype frequencies between cases and controls it was found that carriage of haplotype H (consisting of minor alleles of rs10114470 and rs3810936) exhibited a protective association with CD and UC as its frequency was higher in controls (0.33) compared to cases (0.25) (Table 6). In contrast, haplotypes I and J showed a positive association with CD and UC. The remaining haplotypes did not show any association with CD or UC.

**Table 5:** Structure of haplotypes G-N constructed using LD among all 7 TNFSF15 SNPs examined in the study.

Haplotype	rs10114470	rs3810936	rs6478108	rs4263839	rs6478109	rs7848647	rs7869487
G	C	C					
H	T	T					
I	C	T					
J	T	C					
K			T	G	G	C	T
L			C	A	A	T	C
M			C	A	A	T	T
N			C	G	G	C	C

**Table 6:** Haplotype G-N frequency estimates in controls and IBD patients.

Haplotype	Control	CD	P-value	UC	P-value
G	0.63	0.67	0.11	0.66	0.29
H	0.33	0.25	7.0E – 4*	0.25	3.0E – 4*
I	0.01	0.04	0.01*	0.04	7.0E – 4*
J	0.009	0.03	0.001*	0.04	4.5236E – 5*
K	0.73	0.75	0.28	0.74	0.51
L	0.22	0.19	0.21	0.19	0.23
M	0.03	0.02	0.69	0.03	0.79
N	0.006	0.01	0.10	0.01	0.14

*Indicates significant P value.

## Discussion

TNFSF15 (TL1A), a member of the tumor necrosis factor (TNF) super family, plays a pivotal role in activation and proliferation of T cells [30]. An increased expression level of TNFSF15 in intestinal lamina propria cells correlated with the degree of intestinal inflammation in CD [31]. This was accompanied by cytokine-induced IFN-gamma production by CCR9+ mucosal and gut-homing PB T cells resulting in generation of enhanced Th1 responses and mucosal inflammation [23]. The present study found significant protective associations between SNPs in the *TNFSF15* gene and IBD in the Indian population. While rs3810936, rs10114470 allele and genotype frequencies showed protective associations with both CD and UC, the remaining SNPs showed an association only in terms of genotype frequency suggesting that double dose of the SNP was important in conferring protection.

Of the 7 SNPs that we genotyped in this study, rs3810936 was the only synonymous SNP located in the coding region (exon 4), the remainder being either promoter or intronic polymorphisms. rs3810936 was particularly strongly associated with IBD in the Japanese population. Further studies in two cohorts in the UK confirmed statistically significant associations of this SNP with CD and UC in that population [14]. The rs3810936 CC genotype frequency of 50% in Crohn's disease and 42% in controls noted in our study is very similar to that reported by Tremelling et al in a UK population (49.5% in Crohn's and 42.8% in controls) [18]. Similarly, the rs3810936 TT genotype frequency in a Korean population was 9.2% in cases and 25.4% in controls [19], compared to 8.3% in cases and 13% in controls in the present study.

The SNP rs10114470 has also been significantly associated with Crohn's disease in the Japanese population [27]. In the present study we found significant protective association of the minor allele and the TT genotype with both CD and UC. There is no information on this SNP in IBD in other populations.

The CC genotype of rs6478108 was protectively associated with CD in our study. A similar finding has been noted with CD in the Korean population [19], as well as in the Japanese population [14], [16]. We did not note any association with UC, a finding similar to the lack of association between this SNP and UC in the Japanese [16], although a potential association with Caucasian UC has been reported [14].

The AA genotype of rs4263839 was protectively associated with CD in our study. A meta-analysis of three GWAS also identified the G allele of this SNP as being a risk allele for CD with an OR of 1.22 [21]. A large Italian study confirmed an association of rs4263839 with CD susceptibility in both adult and pediatric IBD populations, and also demonstrated a significant association with early onset UC [22]. It is noteworthy to mention here that a case-control study in the Punjab detected a protective association of the A allele at rs4263839 with UC in that northern Indian population [32]. This could be due to the larger number of participants with UC in that study or due to population-specific differences. The present study comprised participants from southern India and eastern India.

In a GWAS study conducted by Yamazaki et al, rs6478109, rs7848647, rs7869487 were reported to be associated with CD in both Japanese and UK populations, and a potential association was also suggested with Caucasian UC patients [14]. Subsequently these SNPs were replicated in an independent Japanese cohort, showing significant association with CD, but not with UC [16]. Our findings that these variant genotypes of these 3 SNPs were protective against CD but not UC in Indian patients is in concordance with the above. The finding of an association with the mutant homozygous genotype but not with the minor allele implies that homozygosity at this position is necessary for the protective effect. A meta-analysis of 15 GWAS identified 163 IBD loci that met genome wide significance thresholds. TNFSF15 was one of the loci that contributed to both CD and UC [33].

Three common haplotypes (A, B & C) were formed by five TNFSF15 SNPs in Yamazaki's Japanese and Caucasian case-control population [14] and in a Los Angeles based cohort [17]. In order to replicate these findings we did haplotype analysis initially by excluding rs4263839 and rs10114470, since these 2 markers were reported only in later GWAS studies [21], [27]. We were able to reproduce haplotypes A and B in our population with haplotype A establishing risk association with IBD confirmed by carrier frequency of 0.66 in CD, 0.65 in UC and 0.60 in controls. This result was similar to the Japanese population of Yamazaki et al [14]. In contrast, neither Yamazaki's Caucasian case-control group [14] nor Picornell's Caucasian case-control group [17] showed association between haplotype A and CD or UC. A protective association of haplotype B was observed in Japanese, UK and non Jewish Caucasian population [14], [17]. In the present study, a dominant role for the T allele of rs3810936 was noted. In haplotype C, which was protectively associated with IBD in the present study, the only protective allele present in the haplotype was the T allele of rs3810936 and the remaining 4 were all risk alleles, while the haplotype A which was positively associated with IBD contained all the 5 risk alleles for the 5 SNPs.

The haplotype analysis was extended to the two additional SNPs tested in this study. When this was done it became clear that the haplotype should be broken into two blocks, one incorporating rs10114470 and rs3810936, with the remaining 5 SNPs incorporated in another block of haplotypes. The first block containing the two SNPs showed very significant associations with IBD in the Indian population, with haplotype H containing the T alleles of both rs10114470 and rs3810936 showing a very strong protective association with both CD and UC.

Functional analysis of TNFSF15 was not performed in the present study. Nevertheless a prior study carried out in Jewish and non-Jewish CD subjects observed higher expression of TL1A protein on peripheral monocytes in patients carrying haplotype B haplotype compared to haplotype A in Jewish OmpC (E. coli outer membrane porin C) positive subjects suggesting a higher capacity of TL1A-dependent T cell activation and mucosal inflammation in haplotype B positive individuals [34].

In summary, we confirmed strong associations between TNFSF15 polymorphisms and IBD in an ethnically distinct Indian population, and identified haplotypes that protect against both CD and UC. Larger studies will be needed to understand whether there are associations with distinct IBD sub-phenotypes and with disease behaviour in this population.
